# Nimodipine-Induced Blood Pressure Changes Can Predict Delayed Cerebral Ischemia

**DOI:** 10.3389/fneur.2019.01161

**Published:** 2019-10-31

**Authors:** Corinne Fischer, Johannes Goldberg, Sonja Vulcu, Franca Wagner, Daniel Schöni, Nicole Söll, Matthias Hänggi, Jörg Schefold, Christian Fung, Jürgen Beck, Andreas Raabe, Werner J. Z'Graggen

**Affiliations:** ^1^Department of Neurosurgery, Inselspital, Bern University Hospital, University of Bern, Bern, Switzerland; ^2^Institute for Diagnostic and Interventional Neuroradiology, Inselspital, Bern University Hospital, University of Bern, Bern, Switzerland; ^3^Department of Intensive Care Medicine, Inselspital, Bern University Hospital, University of Bern, Bern, Switzerland; ^4^Department of Neurosurgery, University Hospital Freiburg, Freiburg, Germany

**Keywords:** blood pressure variability, cerebral vasospasm, hypertension, delayed cerebral ischemia, cerebral infarction, cerebral perfusion, Nimodipine

## Abstract

**Background:** Early diagnosis of delayed cerebral ischemia (DCI) in patients after aneurysmal subarachnoid hemorrhage (aSAH) still poses a leading problem in neurointensive care. The aim of this study was to analyze the effect of oral Nimodipine administration on systemic blood pressure in patients with evolving DCI compared to patients without DCI.

**Methods:** Systolic (SBP), mean (MAP), and diastolic (DBP) blood pressures were analyzed at the time of Nimodipine administration and additionally 30, 60, and 120 min thereafter on days 1, 3, and 5 after aSAH. Additionally, the 24 h period preceding DCI and in patients without DCI day 10 after aSAH were analyzed. Statistical analysis was performed for SBP, MAP and DBP at time of Nimodipine administration and for the maximal drop in blood pressure after Nimodipine administration.

**Results:** Thirty patients with aSAH were retrospectively analyzed with 17 patients developing DCI (“DCI”) and 13 patients who did not (“Non-DCI”). DCI patients showed a more pronounced rise in MAP and DBP over the examined time period as well as a higher decrease in SBP following Nimodipine administration. A fall of 18 mmHg in SBP after Nimodipine administration showed a sensitivity of 82.4% and specificity of 92.3% for occurrence of DCI.

**Conclusion:** An increase of MAP and DBP after aSAH and a heightened sensitivity to Nimodipine administrations may serve as additional biomarkers for early detection of evolving DCI.

## Introduction

Cerebral vasospasm related ischemia remains the leading cause of disability for patients with aneurysmal subarachnoid hemorrhage (aSAH) who survive the initial bleed and in whom the ruptured aneurysm has been secured (1, 2). Reported incidences of vasospasm-associated cerebral infarction reach up to 44% (3–5). Hence, early detection of evolving cerebral vasospasms and looming misery perfusion is crucial.

Several studies have reported that the L-type calcium channel blocker Nimodipine lowers the incidence of cerebral infarction and poor outcome in aSAH patients (6–9). The British aneurysm Nimodipine trial (1989) showed that poor outcome defined as death or severe disability was reduced by 40% in patients receiving 60 mg Nimodipine orally every 4 h for 21 days or until vasospasm occurred (8). Interestingly, the drug was not shown to lower the incidence of angiographic vasospasm. Therefore, it is hypothesized that the protective effect mainly stems from a reduction of cortical spreading ischemia, a decrease of microthrombi through fibrinolytic activity or neuroprotection (10).

Earlier studies investigating blood pressure changes after aSAH reported a more pronounced blood pressure rise in patients developing severe cerebral vasospasm or DCI (11–13). However, concerning the time point of blood pressure increase the studies report controversial results. Whereas, Faust et al. (11) and Teping et al. (13) found significant blood pressure differences between groups within the first 4 days after the bleeding, Fontana et al. (12) report differences only after day 8. Furthermore, the use of Nimodipine differed between the studies and none of the studies investigated the effect of Nimodipine application on systemic blood pressure.

The aim of this study was to analyze systemic blood pressure effects of orally administered Nimodipine in patients after aSAH who develop clinically symptomatic cerebral vasospasm compared with asymptomatic patients.

## Materials and Methods

This is a single-center retrospective case-control study of patients with aSAH treated at the University Hospital Bern, Bern, Switzerland. Patients aged >18 years and <80 years were included.

### Study Design

Our prospectively conducted database for patients treated with aSAH at the University Hospital Bern was retrospectively searched. Patients hospitalized between July 2012 and October 2015 were included. Inclusion criteria were: (1) patients were treated in the Intensive or Intermediate Care Unit; (2) patients received 60 mg Nimodipine orally every 4 h; (3) no other vasoactive medication or analgosedation was administered after aneurysm treatment; (4) patients had either intra-arterial BP-monitoring or non-invasive BP-monitoring at least every 30 min; (5) patients had a Glasgow Coma Scale (GCS) of >8 after aneurysm treatment (6) patients had no hemodynamic impairments (e.g., preexisting coronary impairments or Takotsubo cardiomyopathy).

Patients were divided into two groups, those who developed DCI (“DCI”) and those who did not (“Non-DCI”). DCI was defined as acute occurring neurological deficit fulfilling the following criteria: (i) decrease of GCS and/or increase of the National Institutes of Health Stroke Scale of at least 2 points for ≥1 h; (ii) exclusion of other causes for neurological deterioration (including epileptic seizure, intracerebral bleeding, hydrocephalus, infection, metabolic causes), and (iii) confirmation of hypoperfusion in a perfusion CT.

### Data Collection

Data was extracted from the institutional electronic Patient Data Management System (Centricity^TM^ Critical Care, General Electric Company, GE Healthcare, United States of America). The system automatically documents all hemodynamic variables in intervals of 2 min. Further data such as clinical scores, fluid balances and administered drugs are entered manually by the bedside team.

Systolic (SBP), mean arterial (MAP), and diastolic (DBP) blood pressure values and heart rate (HR) were assessed at the time point of every single Nimodipine administration (*t*_0_) and additionally 30, 60, and 120 min thereafter. All variables were recorded for the 6 Nimodipine administrations at day 1, 3, and 5 after aneurysm treatment, and Nimodipine administrations 24 h prior to DCI or at day 10 in Non-DCI patients (= *day preceding event*). In addition, GCS and Richmond Agitation Sedation Scale (RASS) scores were assessed at *t*_0_for the above-mentioned time points. The history of preexisting hypertension was directly extracted from the patient medical records.

### Statistical Analysis

For each Nimodipine administration the maximal change of SBP at 30, 60 or 120 min compared to *t*_0_ was calculated. The maximal change was used for further statistical analysis. The same time point was chosen for calculation of MAP, DBP and HR changes. SBP, MAP, DBP, and HR values at *t*_0_, as well as the maximal change after Nimodipine administration were averaged for each analyzed day. GCS and RASS at *t*_0_ were also averaged for each analyzed day.

Statistical analyses were performed using SPSS Statistics 21.0 (IBM, Armonk, NW, USA) and Stata 16 (StataCorp. 2019. Stata Statistical Software: Release 16. College Station, TX: StataCorp LLC.). The Shapiro–Wilk normality test was used to test for normal distribution. Unpaired *t*-test was used to test for significant differences of metric variables between the DCI and Non-DCI groups. Chi-squared test was used to determine potential differences in sex, history of hypertension and method of aneurysm treatment. For hemodynamic variables, GCS and RASS, a mixed effect model for repeated measures was performed to test the interaction between group and days. In addition, an adjusted model for age and gender was performed. Data are presented as mean ± standard deviation (SD) or in figures as mean ± standard error of the mean (SEM). A *p* < 0.05 was considered statistically significant. Diagnostic threshold values for blood pressure changes were calculated using receiver operator characteristics (ROC) curves. From the ROC curves, optimal threshold values to distinguish between patients with and without DCI were derived by seeking the best tradeoff between highest possible sensitivities and specificities.

## Results

### Patient Characteristics

From July 2012 to October 2015, 224 patients were treated for aSAH in the ICU or IMC at the University Hospital of Bern, Bern, Switzerland. One hundred and ninety-four patients were excluded because they did not meet the inclusion criteria. Main reasons for exclusion were either missing blood pressure data for distinct time points or treatment with vasoactive medication. The final study population was composed of 17 patients in the DCI and 13 patients in the Non-DCI group. Table 1 shows clinical characteristics of both patient groups. Although there were no statistical differences between the two groups when comparing age, sex, history of hypertension, method of aneurysm treatment or BNI and Fisher scores, there was a tendency for a higher percentage of female patients in the DCI group. In agreement with other publications, the aneurysms were primarily located in ACOM and PICOM (14).

**Table 1:** Patient characteristics.

	**Non-DCI**	**DCI**	***p*-value**
**Number of Patients**	13	17	
**Median age (years, range)**	56(38–79)	56(36–70)	0.463^#^
**Sex**			0.091^+^
Female	7 (54%)	14 (82%)	
Male	6 (46%)	3 (18%)	
**History of Hypertension**	5 (38%)	5 (29%)	0.602^+^
**BNI Score**			
1	0 (0%)	0 (0%)	
2	4 (31%)	0 (0%)	
3	3 (23%)	5 (47%)	
4	4 (31%)	4 (24%)	
5	2 (15%)	5 (29%)	
**Fisher Score**			
1	0 (0%)	0 (0%)	
2	1 (6%)	0 (0%)	
3	10 (77%)	16 (94%)	
4	2 (15%)	1 (6%)	
**Aneurysm Location**			
ICA	2 (15%)	0 (0%)	
MCA	1 (8%)	2 (12%)	
ACOM	5 (38%)	5 (29%)	
ACA	0 (0%)	0 (0%)	
VA	0 (0%)	2 (12%)	
PICA	1 (8%)	0 (0%)	
SCA	0 (0%)	1 (6%)	
Basilar	0 (0%)	2 (12%)	
A. pericallosa (A2)	1 (8%)	1 (6%)	
PICOM	3 (23%)	4 (24%)	
**Aneurysm Treatment**			
Clipping	1 (8%)	3 (18%)	0.249^+^
Coiling	12 (92%)	13 (76%)	0.427^+^

*Values represent the number of patients with their respective percentages in brackets*.

# *Independent samples t-test*.

+ *Chi-squared test*.

### Hemodynamic Changes at Time of Nimodipine Administration (*t_0_*)

**Figure 1:**
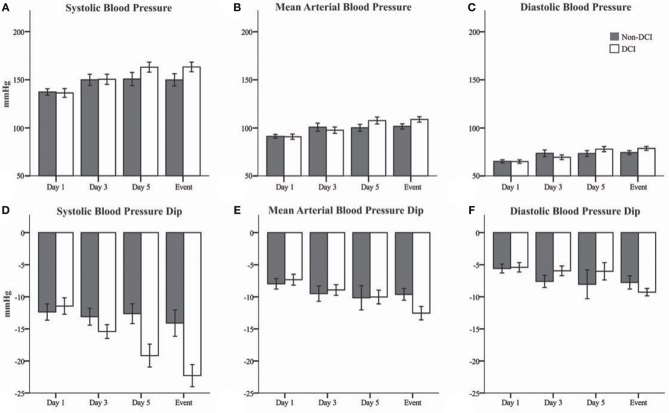
Bar graphs showing systemic blood pressure at *t*_0_ and maximal blood pressure changes after Nimodipine administration in patients with evolving delayed cerebral ischemia (DCI, white) and patients without DCI (Non-DCI, gray). **(A)** Systolic (SBP), **(B)** mean arterial (MAP), and **(C)** diastolic (DBP) blood pressures at time of Nimodipine administration (*t*_0_). **(D)** SBP, **(E)** MAP, and **(F)** DBP changes after Nimodipine administration. Event = day preceding DCI or day 10 in patients without DCI. Mean ± Standard error of the mean.

Blood pressure levels at *t*_0_ increased over the analyzed time period in both patient groups (Figures 1A–C, Table 2). However, the increase of blood pressure was more prominent in patients of the DCI group when compared to patients without DCI. ANOVA for repeated-measures revealed a significant interaction of group^*^time course for MAP (*p* = 0.027) and marginally for DBP (*p* = 0.049) but not for SBP (*p* = 0.092). When adjusting for sex and age, the interaction for SBP also reached significance (*p* = 0.011), whereas DBP was not significant anymore (*p* = 0.061). Adjustment did not change the significance level for MAP (*p* = 0.023). There were no significant changes in HR, GCS, and RASS values over the observation period.

**Table 2:** Systemic blood pressure and heart rate at time of Nimodipine administration (*t*_0_), Nimodipine associated hemodynamic changes, and clinical assessment.

	**Day 1**		**Day 3**		**Day 5**		**Day preceding DCI**		***p*-value**	***p*-value adjusted (sex, age)**
	**Non-DCI**	**DCI**	**Non-DCI**	**DCI**	**Non-DCI**	**DCI**	**Non-DCI**	**DCI**		
SBP (mmHg)	137.3 ± 12.1	136.3 ± 18.7	149.9 ± 21	150.4 ± 21.8	150.7 ± 24.7	163.1 ± 21.6	149.8 ± 23.1	163.4 ± 20.7	0.092	0.011^*^
MAP (mmHg)	91.3 ± 7.1	90.9 ± 11.3	100.8 ± 15.4	97.7 ± 13.5	100.2 ± 12.9	107.7 ± 14.9	101.6 ± 9.7	108.9 ± 11.6	0.027^*^	0.023^*^
DBP (mmHg)	65.2 ± 6.1	65 ± 7.6	73.5 ± 12.8	69.4 ± 10.1	73.3 ± 10.8	78 ± 11.5	74.3 ± 7.1	78.7 ± 8.7	0.049^*^	0.061
HR (bpm)	71.8 ± 11.1	67.9 ± 10.3	73.7 ± 11.5	66.7 ± 11.2	73.9 ± 14	71.3 ± 10.6	77.1 ± 12.4	76.7 ± 12	0.374	0.636
SBP dip (mmHg)	−12.4 ± 4.6	−11.4 ± 5.3	−13.1 ± 4.8	−15.4 ± 4.4	−12.6 ± 5.6	−19.2 ± 7.4	−14.1 ± 7.4	−22.3 ± 7.1	0.037^*^	<0.001^*^
MAP dip (mmHg)	−8 ± 3	−7.3 ± 3.5	−9.5 ± 4.3	−8.9 ± 3.4	−10.1 ± 6.8	−10 ± 4.4	−9.6 ± 3.3	−12.6 ± 4.3	0.169	0.129
DBP dip (mmHg)	−5.6 ± 2.5	−5.4 ± 3	−7.6 ± 3.4	−6 ± 3.1	−8 ± 8.1	−6 ± 5.6	−7.8 ± 3.7	−9.3 ± 2.4	0.427	0.946
HR dip (bpm)	1 ± 3	−1.3 ± 5.2	1.2 ± 4.7	1.8 ± 4.6	1.1 ± 4.3	1.8 ± 4.1	0.4 ± 4.0	0.1 ± 5.6	0.970	0.936
GCS	12.2 ± 2.4	11.9 ± 2.5	13.1 ± 1.4	12.9 ± 1.8	13.5 ± 1.3	13.9 ± 1.1	13.9 ± 1	13.3 ± 1.8	0.162	0.950
RASS	−0.6 ± 1	−1.1 ± 1.3	−0.3 ± 0.8	−0.8 ± 0.9	−0.5 ± 1	−0.2 ± 0.8	0.1 ± 0.8	−0.3 ± 0.9	0.161	0.590

*Values represent means ± Standard deviation*.

* *p ≤ 0.05 DBP, diastolic blood pressure; HR, heart rate; MAP, mean arterial pressure; SBP, systolic blood pressure*.

### Nimodipine-Induced Hemodynamic Changes

In both groups, there was a dip in systemic blood pressure after Nimodipine application (Figures 1D–F, Table 2). However, the dip in blood pressure became more prominent during the course of disease in patients developing DCI compared to patients who did not. This effect was most prominent for SBP (*p* = 0.037). After correction for sex and age, this effect proved to be even more prominent (*p* < 0.001). HR remained unchanged in both groups after Nimodipine application.

### Threshold Values for Predicting DCI

Table 3 shows diagnostic threshold values for MAP and DBP changes at *t*_0_ as well as for Nimodipine induced SBP dips. A Nimodipine induced SBP drop of −18 mmHg showed the highest sensitivity and specificity for predicting DCI.

**Table 3:** AUCs with corresponding 95% CIs of changes in mean and diastolic blood pressure at *t*_0_ and drops of systolic pressure after Nimodipine administration, as well as sensitivities and specificities for the detection of DCI.

	**AUC (95% CI)**	**Threshold (mmHg)**	**Sensitivity**	**Specificity**
MAP change	0.719 (0.524–0.915)	15	0.765	0.769
DBP change	0.719 (0.524–0.915)	11	0.882	0.615
SBP dip	0.799 (0.614–0.983)	−18	0.824	0.923

*AUC, area under the curve; CI, confidence interval; DBP, diastolic blood pressure; MAP, mean arterial pressure; SBP, systolic blood pressure*.

## Discussion

In this retrospective study we analyzed systemic blood pressure changes in patients with aSAH after aneurysm treatment receiving Nimodipine orally every 4 h. At time of Nimodipine administration (*t*_0_), MAP, and DBP increased significantly in patients developing DCI when compared to clinically asymptomatic patients. Additionally, a heightened responsiveness of SBP to Nimodipine in patients developing DCI was found.

### Systemic Blood Pressure Rise at Time of Nimodipine Administration (*t*_0_)

Three previous studies already investigated systemic blood pressure changes after aSAH. All reported a more pronounced systemic blood pressure increase in patients developing severe angiographic vasospasm or DCI (11–13). However, the time point of blood pressure increase differed between these studies. Whereas, Faust et al. (11) and Teping et al. (13) reported a significant blood pressure increase in patients developing severe vasospasms or DCI already within the first 4 days after bleeding, Fontana et al. (12) found differences only after day 8. Only the studies by Fontana et al. (12) and Teping et al. (13) included patients treated with Nimodipine but did not investigate the effect of Nimodipine application on blood pressure. We only enrolled patients who received 60 mg Nimodipine orally every 4 h and used a clinical endpoint for DCI. In parallel to the earlier studies, our study also shows an increase of systemic blood pressure after aSAH, which was more pronounced for MAP and SBP in patients who develop DCI. The findings of the previous and our own study suggest an association between development of cerebral vasospasms or DCI and spontaneous blood pressure rise. In fact, it has been shown that sympathetic activation increases after aSAH in patients with evolving cerebral vasospasms (15, 16). To maintain cerebral blood flow constant in the face of narrowing cerebral vessels, the brain has to increase cerebral perfusion pressure. This is mediated by sympathetic activation and rise of endogenous catecholamine release leading to an increase of peripheral resistance and therefore a rise of MAP and cerebral perfusion pressure, respectively (17). The smaller increase of MAP and DBP in the Non-DCI group is most likely due to milder sympathetic activation because of less severe, clinically not relevant vasospasms.

### Drop in Systemic Blood Pressure After Nimodipine Administration

The L-type calcium channel blocker Nimodipine has a strong vasodilating effect. Interestingly, SBP drops after Nimodipine application more pronounced in patients with evolving DCI. Earlier studies showed contradictory effects of Nimodipine on catecholamine action and sympathetic nervous system tone (18–23). It has been postulated that Nimodipine affects norepinephrine (NE) mediated contraction of blood vessels, postsynaptic adrenoceptors and lowers plasma catecholamine concentrations. One hypothesis for the more prominent dip of SBP in patients with evolving DCI might be that DCI patients experience a higher sympathetic nervous tonus and a surge in NE to sustain brain tissue perfusion. Therefore, they have likely reached maximum capacity of their endogenous NE levels and are subsequently more susceptible to the effect of Nimodipine (16, 24, 25). It is still unclear why the blood pressure drop was significant for SBP but not for MAP and DBP. A possible explanation was reported by Van Meel et al. (20) who showed in a rat model that Nimodipine helped to block out the effects of NE on SBP but not on DBP. Hence, patients with impending DCI and consequently higher endogenous NE levels would therefore show a greater fall in SBP than patients without DCI in response to Nimodipine. The effect of NE on DBP would remain uninfluenced by Nimodipine, resulting in non-significant blood pressure differences in MAP and DBP between the two groups. In a recent paper published by Kieninger et al. (26) studying the incidence of arterial hypotension in patients treated with Nimodipine after aSAH and perimesencephalic SAH, episodes of hypotension after Nimodipine administration were predominantly found if the drug was given orally in patients with higher-grade SAH. Furthermore, an association was found between a lower risk of unfavorable outcome and patients who showed less episodes of hypotension after Nimodipine administration. As DCI is the leading cause of unfavorable outcome in patients with SAH who survive the initial bleed, the findings of the study by Kieninger et al. indirectly support the findings of our study. However, contrary to the present study, Nimodipine dosages were reduced or stopped on over 40% of the examined days, whereas in our analysis, only patients receiving the full Nimodipine dosages were enrolled.

There have been other studies analyzing blood pressure variability in relationship with different outcome measurements after aSAH (27–31). Even though all of those studies established a higher blood pressure variability to be associated with worse scores in the examined parameters, their results cannot be directly compared with those of the present study due to different study designs.

### Prediction of DCI in Clinical Practice

Our data shows that distinct changes of systemic blood pressure have sensitivities and specificities as high as 88.2 and 92.3%, respectively for prediction of DCI. Reported sensitivities and specificities for imaging are comparable (transcranial Doppler: sensitivity 90%, specificity 71%; CT perfusion imaging: sensitivity 74–93%, specificity 63–93%) (32–40) or even lower (CT angiography: sensitivity 64%, specificity 50%) (35). Hence, bedside pattern recognition of evolving systemic blood pressure alterations may serve as an easy clinical tool for early detection of looming DCI in patients after aSAH which complements imaging. The use of an electronic patient data management system supports pattern recognition.

### Limitations

The retrospective and single center study design represents the major limitation of this study. Additionally, only a small cohort of all patients treated for aSAH in our hospital could finally be enrolled into the study because of the very restrictive inclusion criteria. The major reasons for exclusion of patients were missing blood pressure data points or treatment with vasoactive medication (either catecholamines of blood pressure lowering drugs) after securing the aneurysm. In our opinion the use of well-defined and restrictive inclusion criteria bears the advantage of minimizing the natural bias of a retrospective study design to some extent. The findings of this study have to be verified with a prospective study design. Furthermore, the calculated threshold values for prediction of DCI also need to be verified with a prospective study.

## Conclusion

Our results show that an increase of systemic blood pressure after aSAH and a heightened sensitivity to Nimodipine administrations may serve as additional biomarkers for early detection of evolving DCI. Especially, SBP drops after Nimodipine administration, along with an overall rise in MAP and DBP can point toward an increased risk for DCI.

## Data Availability Statement

The datasets generated for this study are available on request to the corresponding author.

## Ethics Statement

This study was carried out in accordance with the recommendations of the local ethics committee (Kantonale Ethikkommission Bern, Switzerland) with written informed consent from all subjects. All subjects gave written informed consent in accordance with the Declaration of Helsinki. The protocol was approved by the institutional review board of the University Hospital Bern and the local ethics committee (Kantonale Ethikkommission Bern, Switzerland).

## Author Contributions

WZ'G, JB, AR, MH, and JS contributed to conception and design of the study. CFi, JG, SV, FW, DS, NS, and CFu collected the data. CFi and WZ'G performed the statistical analysis. All authors interpreted the results. CFi wrote the first draft of the manuscript. WZ'G, JS, MH, and JB wrote sections of the manuscript. All authors contributed to manuscript revision, read, and approved the submitted version and provided the approval for publication of the content and agree to be accountable for all aspects of the work in ensuring that questions related to the accuracy or integrity of any part of the work are appropriately investigated and resolved.

### Conflict of Interest

The authors declare that the research was conducted in the absence of any commercial or financial relationships that could be construed as a potential conflict of interest.

## Abbreviations

ANOVA: Analysis of Variance
aSAH: Aneurysmal Subarachnoid Hemorrhage
CPP: Cerebral Perfusion Pressure
DBP: Diastolic Blood Pressure
DCI: Delayed Cerebral Ischemia
GCS: Glasgow Coma Scale
HR: Heart rate
ICU: Intensive Care Unit
IMC: Intermediate Care Unit
MAP: Mean Arterial Pressure
NE: Norepinephrine
NIHSS: National Institutes of Health Stroke Scale
RASS: Richmond Agitation Sedation Scale
ROC: Receiver-Operating Characteristic
SAH: Subarachnoid Hemorrhage
SBP: Systolic Blood Pressure.
